# *Plasmodium falciparum* full life cycle and *Plasmodium ovale* liver stages in humanized mice

**DOI:** 10.1038/ncomms8690

**Published:** 2015-07-24

**Authors:** Valérie Soulard, Henriette Bosson-Vanga, Audrey Lorthiois, Clémentine Roucher, Jean- François Franetich, Gigliola Zanghi, Mallaury Bordessoulles, Maurel Tefit, Marc Thellier, Serban Morosan, Gilles Le Naour, Frédérique Capron, Hiroshi Suemizu, Georges Snounou, Alicia Moreno-Sabater, Dominique Mazier

**Affiliations:** 1 Sorbonne Universités, UPMC Univ Paris 06, CR7, Centre d'Immunologie et des Maladies Infectieuses (CIMI-Paris), 91 Bd de l'hôpital, F-75013 Paris, France; 2 INSERM, U1135, CIMI-PARIS, 91 Bd de l'hôpital, F-75013 Paris, France; 3 CNRS, ERL 8255, CIMI-PARIS, 91 Bd de l'hôpital, F-75013 Paris, France; 4 Université FHB, UFR SPB, Departement de Parasitologie-Mycologie, BP V 34 Abidjan, Ivory Coast; 5 AP-HP, Groupe Hospitalier Pitié-Salpêtrière, Service Parasitologie-Mycologie, Centre National de Référence du Paludisme, 83 Bd de l'hôpital, F-75013 Paris, France; 6 UPMC Univ. Paris 06, INSERM, UMS28, 105 Bd de l'hôpital, F-75013 Paris, France; 7 AP-HP, UPMC Univ. Paris 06, Groupe Hospitalier Pitié-Salpêtrière, Service d'anatomie et cytologie pathologiques, 83 Bd de l'hôpital, F-75013 Paris, France; 8 Central Institute for Experimental Animal, Kawasaki, Kanegawa, Japan

## Abstract

Experimental studies of *Plasmodium* parasites that infect humans are restricted by their host specificity. Humanized mice offer a means to overcome this and further provide the opportunity to observe the parasites *in vivo*. Here we improve on previous protocols to achieve efficient double engraftment of TK-NOG mice by human primary hepatocytes and red blood cells. Thus, we obtain the complete hepatic development of *P. falciparum*, the transition to the erythrocytic stages, their subsequent multiplication, and the appearance of mature gametocytes over an extended period of observation. Furthermore, using sporozoites derived from two *P. ovale*-infected patients, we show that human hepatocytes engrafted in TK-NOG mice sustain maturation of the liver stages, and the presence of late-developing schizonts indicate the eventual activation of quiescent parasites. Thus, TK-NOG mice are highly suited for *in vivo* observations on the *Plasmodium* species of humans.

## Abstract (web summary)

Mice engrafted with human cells are useful models for research on human malaria parasites. Here the authors show that the complete life cycle of *Plasmodium falciparum* and the liver stages of *Plasmodium ovale* can be studied in mice doubly engrafted with human primary hepatocytes and red blood cells.

## Graphical abstract

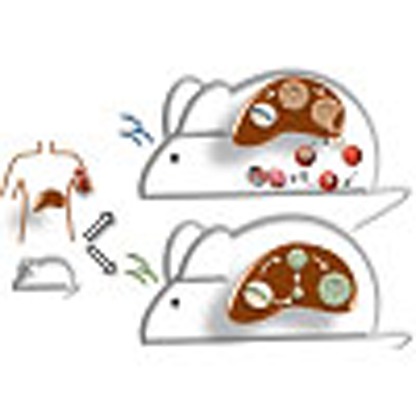

Malaria remains a major cause of death and morbidity worldwide^1^, with infections by *Plasmodium falciparum* accounting for the majority of malaria mortality, though the less virulent *P. vivax*, and probably *P. ovale*, also contribute significantly to morbidity.

*Plasmodium* sporozoites injected by an infected mosquito migrate to the liver and initiate the hepatic stage of the parasite life cycle by invading hepatocytes within which they multiply and differentiate into schizonts containing thousands of hepatic merozoites. These merozoites are subsequently released into the blood where they initiate the erythrocytic stage by invading and replicating within red blood cells (RBCs). Some of these asexual blood parasites differentiate into gametocytes that will ensure parasite transmission to the mosquito vector. *P. vivax* and *P. ovale* show a slightly different life cycle within the mammalian host, as some sporozoites once in the liver do not develop immediately into schizonts, but remain at an uninucleate stage, in a quiescent form named hypnozoite, before resuming hepatic development on the impulse of still unknown factors, causing relapses weeks, months or even years after the primary infection^2^.

The search for novel or improved means to control malaria, whether chemotherapeutic or immunoprophylactic, is dependent on the availability of practical experimental models for preclinical investigations. Investigations on the parasite species that infect humans are hampered by the strict specificity to the host's cells. *In vivo* models have been limited to infections of selected species of South American primates, that are now increasingly restricted by ethical and cost considerations. At present, routine *in vitro* cultivation is only available for the blood stages of *P. falciparum*, and that of the liver stages of this parasite is best obtained using primary human hepatocytes alone or in coculture^3^,^4^,^5^, though hepatic development in HCO4 cell lines has been reported albeit with much lower levels of infection^6^,^7^. Cultivation of the *P. vivax* liver stages is equally well sustained when human primary hepatocytes or a HepG2 cell line are used^8^,^9^.

In general, *Plasmodium* hepatic stage cultures are limited to 5–10 days of cultivation, though recent advances have extended this to a month or so^3^,^4^. Consequently, our knowledge of the biology and immunology of the malarial liver stages is limited and fragmentary, and is minimal for the hypnozoites. The major interest in these hepatic forms is that they represent the initial obligatory phase of the life cycle of *Plasmodium* in the human host. During this pre-erythrocytic phase the parasites are present in very low numbers and generally develop over a short period (5–14 days). This makes them an ideal target for parasite elimination^10^.

Immunodeficient mice engrafted with human cells, offer a cost-effective and easily manipulated laboratory model to study human-restricted pathogens *in vivo*^11^. To date, such mice engrafted with human hepatocytes (hHEP) or human RBCs (hRBC) have been shown to sustain *P. falciparum* hepatic development and the multiplication of the blood stages^12^,^13^,^14^,^15^,^16^,^17^. Efficient liver humanization relies simultaneously on an immunodeficiency in the host to facilitate xenotransplantation and on the selective elimination of endogenous murine hepatocytes to make room for the transplanted human hepatocytes to repopulate the liver. In this manner, *P. falciparum* liver stage maturation was first observed in homozygous Alb–UpA SCID mice^13^,^14^, where expression of the hepatotoxic urokinase plasminogen activator (UpA) transgene under the albumin promoter leads to a constitutive loss of endogenous hepatocytes. Thus, UpA–SCID mice are best engrafted at very young age (3–4 weeks) but given their low level of immunodeficiency additional treatment to deplete NK cells and macrophages is required^13^. Recently, mice deficient for fumarylacetoacetate hydrolase (FAH^−/−^) with the broader immunodeficient Rag2^−/−^ IL2Rγ^−/−^ background (FRG) were also used for liver humanization^18^. The FAH^−/−^ mice suffer from an acute liver failure that can be rescued by providing NTBC (2-(2-nitro-4-trifluoromethylbenzoyl)-1,3-cyclohexanedione) at regular intervals pre- and post-human hepatocytes transplantation. The FRG mice backcrossed onto the NOD background (FRG NOD) allowed full maturation of the *P. falciparum* liver stages up to the generation of infectious hepatic merozoites^19^. Here we have used TK-NOG mice that express the HSVtk transgene under the albumin promoter onto the NOD SCID IL2Rγ^−/−^ background^20^. In this mouse strain, the loss of endogenous hepatocytes is inducible by a brief exposure to a non-toxic dose of gancyclovir, a method that is rapid and temporally restricted, and routinely leads to subtantial hHEP repopulation (60–80%). These levels are comparable to those obtained in UpA–SCID and FRG mice^13^,^14^,^19^, and more importantly, they are maintained without the need for any additional treatment. Furthermore, similarly to FRG NOD mice, carriage of the SIRPα gene NOD allele prevents recognition of CD47 on the transplanted human cells, which highly facilitates engraftment with hRBC^21^.

The aim of our studies was to exploit the TK-NOG mice to obtain animals simultaneously engrafted with hHEP and and hRBC, and to ascertain whether they could then sustain the full *P. falciparum* life cycle, from sporozoite inoculation to gametocyte production. During the course of the study, we also had the opportunity to infect the mice with *P. ovale* sporozoites and to follow-up the infection for a sufficiently long period to suggest the presence of hypnozoites.

## Results

### *P. falciparum* hepatic development in TK-NOG mice

**Figure 1:**
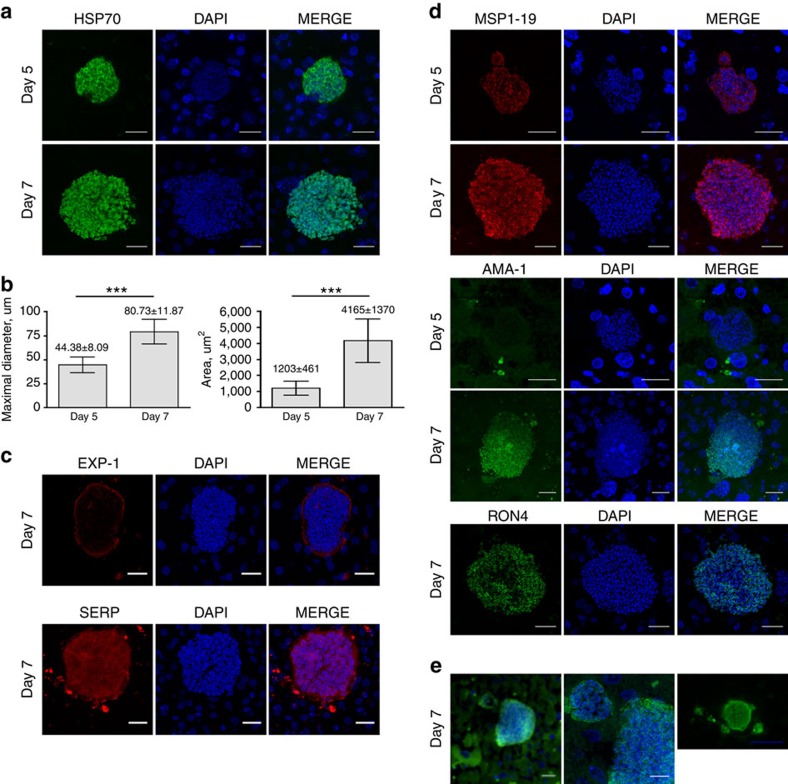
Hepatic development of *P. falciparum* in LH TK-NOG mice. Representative pictures of *P. falciparum* schizonts at days 5 and 7 post-infection. Sixteen-μm thick frozen and 5-μm thick deparaffinized liver sections were stained by indirect immunofluorescence with antibodies specific to *P. falciparum* and DAPI to stain host cell and parasite nuclei. (**a**). Day-5 and day-7 schizonts stained for *Plasmodium* HSP70, scale bar, 20 μm. (**b**). Maximal diameter and area of day-5 and day-7 schizonts; *n*=32 at day 5 and *n*=27 at day 7, both from 3 different lobes, mean±s.d. ***P<0.0001, Mann–Whitney *U*-test. (**c**). Day-7 schizonts stained for EXP-1 and SERP. (**d**). Schizonts were stained for MSP-1, AMA-1 and RON-4 at day 5 and day 7. Scale bar, 20 μm. (**e**). Merosome-like structures stained for HSP70 and DAPI observed in day-7 schizonts, left picture, Scale bar, 20 μm; central picture, magnification of left picture showing merozoites nuclei in the merosome-like structure, Scale bar, 10 μm; right picture, Scale bar, 50 μm.

Liver-humanized (LH) TK-NOG mice were inoculated with 1–3 million *P. falciparum* sporozoites, killed at different time points thereafter, and their livers were analysed for the presence of parasites by immunofluorescent staining of *Plasmodium* HSP70 (Fig. 1a). Human albumin (hAlb) plasma levels on the day of sporozoites inoculation varied between the mice (2–10 mg ml^−1^). In the mice where hALb levels were approximately 5 mg ml^−1^ (liver humanization≥60%, *n*=2) around 20 parasite forms could be observed in each 40–45 mm^2^ liver section obtained on day 7 after inoculation with 3 million *P. falciparum* sporozoites (Supplementary Fig. 1a–b).

Hepatic schizonts were observed in liver sections made on day 5 and day 7 post-infection (Fig. 1a), with clearly individualized merozoites distinguishable in the mature day-7 schizonts. The diameter of the maturing liver forms ranged from 30.5 to 59.72 μm (*n*=32, mean±s.d.=44.38±8.09 μm) for the day 5 parasites, and from 60.2 to 105 μm (*n*=27, mean±s.d.=80.73±11.87 um) for the day 7 parasites (Fig. 1b). These values are the double of those usually observed at equivalent time points in *in vitro*-cultured *P. falciparum*-infected primary human hepatocytes^5^,^22^. The parasites' mean area increased from 1203±461 μm^2^ on day 5 to 4165±1370 μm^2^ on day 7. We also confirmed that the parasites developed exclusively in the engrafted hHEP stained for hAlb (Supplementary Fig. 2). It was interesting to note that by contrast to *in vitro* observations of *P. falciparum* in primary human hepatocytes cultures^23^, uninucleate forms were not observed in the liver sections analysed.

To facilitate future investigations, we set up a medium throughput analysis using a fluorescent slide scanner (Nanozoomer) to screen liver sections. Identification, measurement and quantification of day 7 *P. falciparum* schizonts were thereby rapid and accurate (Supplementary Fig. 1a,b).

Maturation of the liver forms was assessed by a panel of antibodies raised against different parasite proteins expressed at different stages of development. Day-7 schizonts in sections stained for *P. falciparum* Exported Protein-1 (*Pf*EXP-1, ref. 24), a mid to late liver stage antigen, showed circumferential staining consistent with its expected localization at the parasitophorous vacuole membrane (Fig. 1c, upper panel). We also noted that the serine-rich protein (SERP, ref. 25), a soluble protein secreted in the lumen of the parasitophorous vacuole in blood-stage parasites^26^, was also found in the parasitophorous vacuole of the day-7 hepatic parasites (Fig. 1c, lower panel). Day-5 hepatic parasites stained positively only for the Merozoite Surface Protein 1-p19 (MSP1_19_), whereas day-7 parasites additionally stained positively for the Apical Merozoite Antigen-1 (AMA-1, ref. 27) and the Rhoptry Neck Protein 4 (RON-4, ref. 28) (Fig. 1d). These results are consistent with a normal hepatic maturation in LH TK-NOG mice culminating in mature merozoites capable of invading RBC. This conclusion was further re-enforced by observation of merosome-like structures in the liver of day-6- and day-7-infected LH TK-NOG mice (Fig. 1e). Merosomes, vesicles containing packets of merozoites, have been described for *Plasmodium* species that infect rodents as the means by which hepatic merozoites reach the blood stream *in vivo*^29^,^30^.

### *P. falciparum* blood stages in sporozoite-infected TK-NOG mice

Having established that TK-NOG mice sustain full maturation of the *P. falciparum* hepatic stages, we wished to establish whether the liver-to-blood transition could take place in these animals. It had been previously shown that NOG mice can be efficiently engrafted with hRBC and can sustain the multiplication of *P. falciparum* blood stages^16^,^31^. We first established that TK-NOG mice could also be humanized with hRBC through daily intraperitoneal (i.p.) injection of hRBC and without further immunosuppressive treatment, as described in NOD^*SCID/β2m*−/−^ mice^15^. Thus, we obtained 50–60% hRBC chimerism within 6 days, and 80–99% by day 12. These levels remained at or above 80% thereafter in all the engrafted mice (Supplementary Fig. 3). We further obtained similar results in TK-NOG mice that had been previously engrafted with hHEP, and in these mice the high levels of hRBC chimerism was maintained for up to 5 weeks (Supplementary Fig. 3).

**Figure 2:**
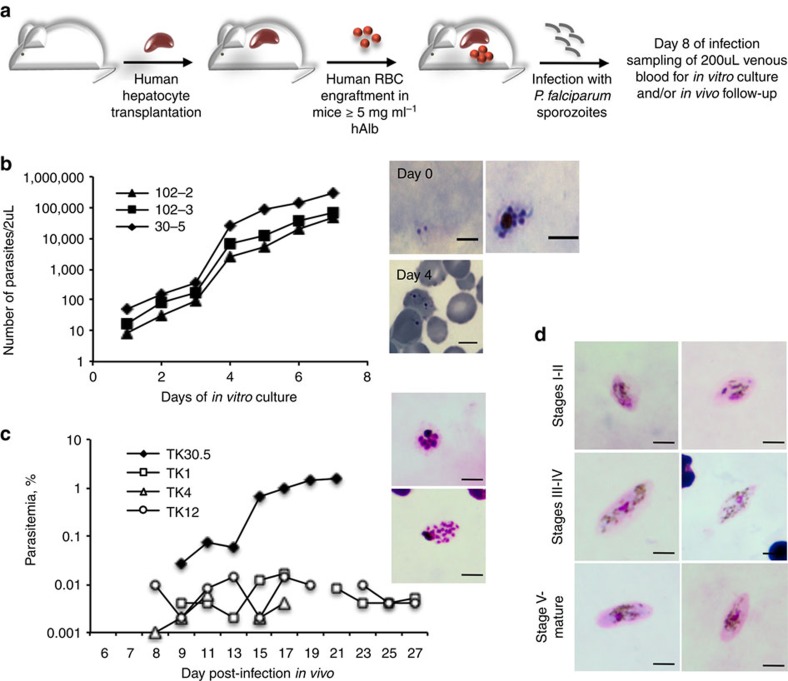
Asexual and sexual blood stages of *P. falciparum* in sporozoite-infected TK-NOG mice engrafted with human hepatocytes and RBC. (**a**). Experimental procedure for the double engraftment of TK-NOG mice with human hepatocytes and RBC before infection with *P. falciparum* sporozoites. (**b**). *In vitro* asexual blood stage culture: at day 8 of infection, 200 ul of venous blood was put in culture to follow emergence of parasitaemia. Results of one experiment with three mice are expressed as the number of parasites within 2 μl of blood for each mouse. Results are representative of three independent experiments performed with a total of eight mice. Representative pictures of a trophozoite and a schizont observed on Giemsa-stained thick blood smears at day 0 of *in vitro* culture (= day 8 of infection) and of trophozoites observed on Giemsa-stained thin blood smears at day 4 of culture. (**c**). *In vivo* asexual blood stage development: individual parasitaemias were determined using Giemsa-stained thin blood smears from a total of 4 mice from two independent experiments. Representative pictures of schizonts observed on Giemsa-stained thick blood smears during the course of infection, scale bar, 5 μm. (**d**). *In vivo* sexual blood stage development: representative pictures of gametocytes at different stages of their development as observed in Giemsa-stained thick blood smears from two mice.

Groups of TK-NOG mice were then first engrafted with hHEP and when plasma hAlb levels reached 5.0–9.8 mg ml^−1^ (liver humanization≥60%), they were injected with hRBC daily for 6 days, before inoculation with 1.75 × 10^6^ to 3.5 × 10^6^ *P. falciparum* sporozoites. Daily injections of hRBC were continued until the end of the observations. In this manner, optimal levels of hRBC (≥80%) were present in the mice at the time (day 6) when the hepatic schizonts are expected to reach full maturity and release infective merozoites (Supplementary Table 1). At day-8 post-sporozoite inoculation a sample of 200 μl of peripheral mouse blood was collected and used to initiate *in vitro* cultures that were then followed over the next 8 days to assess for the presence of *P. falciparum* blood stage parasites (Fig. 2a). In three independent experiments, cultured blood from all eight mice showed parasites within the first 4 days of *in vitro* culture (Fig. 2b). The robustness of the protocol is further underlined by the fact that three distinct batches of hHEP and of sporozoites (though all from the same line, NF54) were used for these experiments.

Furthermore, the parasitaemia was monitored in thin and thick tail-blood smears collected daily in four of the mice above (from two independent experiments) starting from day-8 post-sporozoite inoculation. In all four mice, a patent parasitaemia was detected. In the first experiment, one mouse became positive from day 9 with the parasitaemia increasing to 1.52% by day 21 at which time the mouse was killed (Fig. 2c). In the second experiment, parasites were first detectable on day 8 post-sporozoite injection in all three mice but the parasitaemia remained low, never exceeding 0.016%, throughout the 4 weeks of follow-up (Fig. 2c). The reason for the difference in peak parasitaemia levels in the two experiments might be related to batch-to-batch variations in human primary hepatocyte receptivity or sporozoite infectivity (unpublished observations and ref. 4). Furthermore, two of the four mice above were monitored for gametocyte appearance and, in both mice, gametocytes were detected at day-26 post-sporozoite injection, with immature stages I–IV observed first and then with the progressive appearance of stage V mature gametocytes (Fig. 2d). Gametocytaemia was maintained thereafter, during the two additional weeks of follow-up, with the maximum proportion of total and mature gametocytes observed reaching 8.5% and 2.5% of the circulating parasites, respectively.

### LH-TK-NOG mice are suitable for investigations on a relapsing malaria parasite

We had the opportunity to investigate the suitability of the TK-NOG mice for the study of *P. ovale*, a parasite prevalent in sub-tropical Africa, through the availability of two blood samples obtained from different patients with *P. ovale* infection. The sporozoites obtained from *Anopheles stephensi* mosquitoes that had fed on the *P. ovale*-infected blood samples, were used to inoculate LH TK-NOG mice.

Given that *P. ovale* liver stage maturation has been reported to take 9 days in humans^32^, we assessed the parasite's development in some of the TK-NOG mice on day 8 post-sporozoite inoculation, to search for mature schizonts and hypnozoites, and in other mice on day 21, with the aim to seek evidence for the presence of late-developing schizonts that would result from hypnozoite activation.

**Figure 3:**
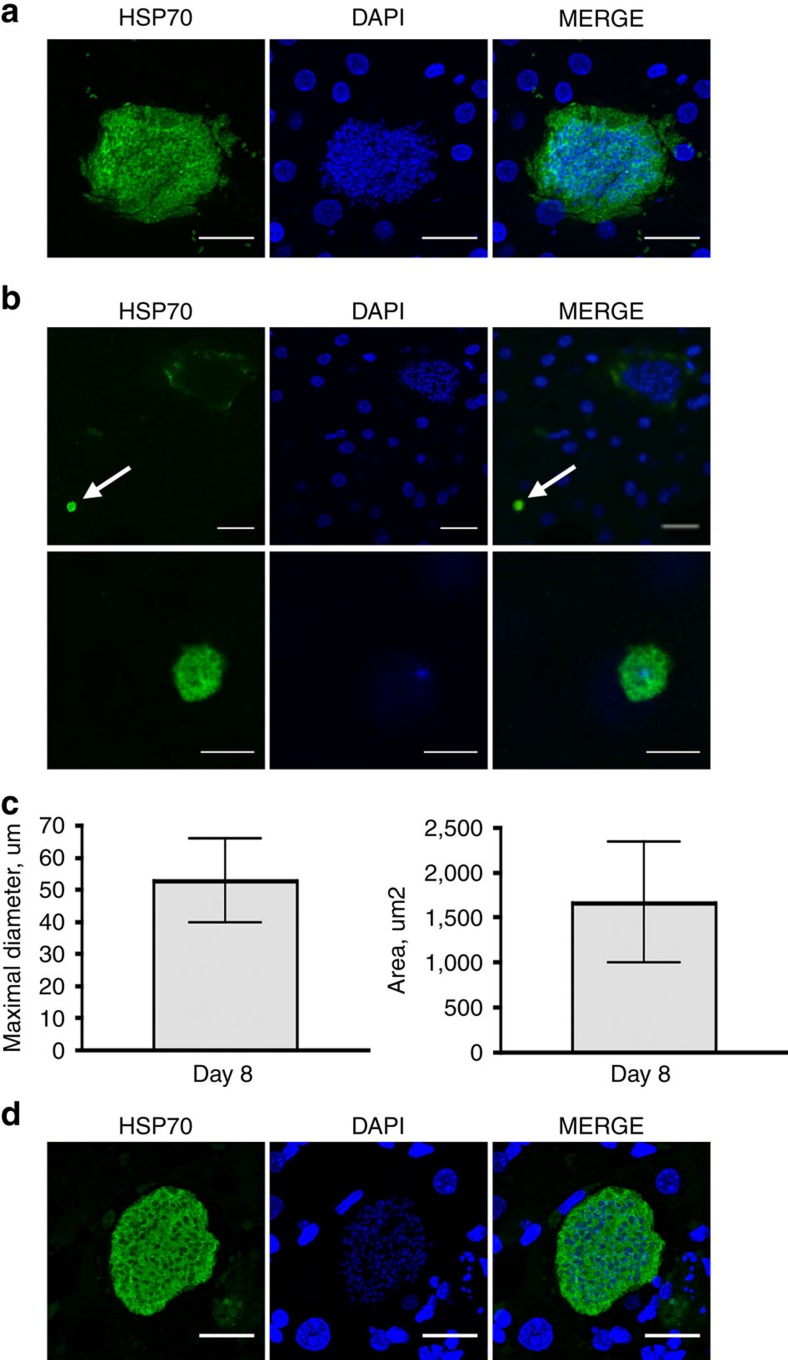
Hepatic development of *P. ovale* in LH-TK-NOG mice. Representative pictures of *P. ovale* schizonts and hypnozoites at days 8 and 21 post-infection. 50-μm thick frozen (**a**,**b**) or 5-μm thick deparaffinized liver sections (**d**) were stained by indirect immunofluorescence with immune serum specific to *Plasmodium* HSP70 and DAPI to stain host cell and parasite nuclei. (**a**). Day-8 schizonts, scale bar, 20 μm. (**b**). Upper panel: day-8 schizont and—hypnozoite (white arrow), scale bar, 20 μm. Lower panel: higher magnification of the hypnozoite showing the single nucleus within the 5 μm round-shape parasite, scale bar, 5 μm. (**c**) Maximal diameter and area of day-8 schizonts; *n*=35, from three different lobes, mean±s.d. (**d**). Day-21 schizont, scale bar, 20 μm.

In a first experiment, 2 mice having 6.0 and 6.9 mg ml^−1^ hAlb plasma levels were infected with 280,000 sporozoites. Large mature *P. ovale* schizonts were readily detectable in the engrafted hHEP at day-8 post-sporozoite inoculation (upper panel, Fig. 3a, Supplementary Fig. 4a). Their median±s.d. diameter was 52.63±13.01 μm and areas 1664±671 μm^2^ (*n*=35, Fig. 3c). In this experiment numerous developing schizonts were observed in liver sections (15–20 schizonts per 30–35 mm^2^ liver section) and were easily identified using the fluorescent slide scanner (Supplementary Fig. 4b). We also observed small, round-shaped uninucleate parasites with a diameter of ∼5 μm (Fig. 3b, Supplementary Fig. 4b, lower panel) that could be hypnozoites, and that represented 15% of the total number of forms observed on day 8 (10 of the 65 forms detected in thick serial liver sections from two different lobes). Finally, we observed a single hepatic schizont, of 40-μm diameter, among 123 sections from four pieces of different lobes analysed from a mouse sacrificed on day-21 post-sporozoite injection (Supplementary Fig. 4c), the only late parasite form that we could detect in this experiment. In a second infection with 200,000 sporozoites from another clinical isolate that were inoculated to two LH TK-NOG mice (1 and 1.7 mg ml^−1^ plasma hAlb), fewer numbers of schizonts and uninucleate hypnozoite-like forms were observed on day 8, likely due to the low level of liver humanization of the mouse, but several schizonts were observed at day 21 (Fig. 3d). In neither experiment were any small forms observed on day 21. This suggests that all parasites had resumed their hepatic development or that the number of persisting quiescent parasites were too low to be detected. It should be noted that small forms were not observed in *P. falciparum*-infected mice sacrificed on day 5 or day-7 post-sporozoite inoculation, nor could any parasites forms be found in the liver of a *P. falciparum*-infected mouse sacrificed on day 21 post-infection.

## Discussion

We describe the successful double engraftment of TK-NOG mice with hHEP and hRBC, with efficiencies of ‘humanization' reaching up to 90% for both cell types. In all the mice, the engrafted hHEP were receptive to infection by sporozoites from two of the species that infect humans, *P. falciparum* and *P. ovale*, and sustained maturation of the resulting hepatic stages.

Observations of the *P. ovale* hepatic stages are very few in number indeed. *In vivo* observations are restricted to a single occasion in humans^32^, and apart from one case where *Saimiri* monkeys were used^33^, the remainder were principally made on infected chimpanzees^34^,^35^. *In vitro* observations were also made only once using primary human hepatocytes^36^ and once using primary *Saimiri* hepatocytes^33^. Experimental infections with *P. ovale* were last made in humans in the mid 1960s when five volunteers were inoculated with sporozoites^37^, and thereafter using chimpanzees. West African strains of the parasites were mainly used for these observations. The *P. ovale* sporozoites used in the current study were derived from two distinct *P. ovale* clinical isolates, collected 9 months apart from patients who acquired the infection in Africa. These sporozoites proved to be infectious to human primary hepatocytes engrafted in TK-NOG mice, where they developed into mature hepatic schizonts of a size consistent with those observed in the human liver^32^. Despite the relatively low sporozoite inoculum (250,000 and 300,000), the resulting *P. ovale* hepatic forms were sufficiently numerous to allow easy detection in the liver sections screened 8 days following inoculation. Along these mature schizonts, we observed small round-shaped uninucleate parasite forms of ∼5 μm in diameter. Furthermore, maturing schizonts were observed in liver sections taken on day-21 post-infection. The fact that the *P. ovale* hepatic stages normally complete their maturation in 9 days^32^ and that early relapses can occur within the first month after the infective mosquito bite^38^, are consistent with a hypnozoite origin of the maturing forms observed on day 21. This is also supported by observations in TK-NOG mice inoculated with sporozoites of a non-relapsing species, *P. falciparum*, where neither small uninucleate forms could be observed on day 5 or 7 liver sections, nor any parasite forms could be found on those made on day 21. Nonetheless, conclusive proof of the occurrence of hypnozoites in TK-NOG mice awaits further observations.

For *P. falciparum*, we could demonstrate a successful transition from the hepatic to the erythrocytic stages of the infection, as all the mice inoculated with sporozoites developed a patent blood parasitaemia, which was maintained over 3–6 weeks of follow-up. It was further noted that *P. falciparum* gametocytogenesis culminating in the production of mature gametocytes was also observed, thus providing a laboratory mice model that recapitulates the complete life cycle of *P. falciparum.* Complete development of the *P. falciparum* hepatic stages had been previously obtained in immunocompromised mice. This was reported in Alb–UpA SCID mice by one group^14^ but not by the other^13^, and more recently in FRG mice^19^. In these mice, treatment to maintain optimal conditions for engraftment had been administered, though this did not appear to have influenced parasite development. In these mice as well as the TK-NOG described here, the diameters of the hepatic schizonts on day-7 post-inoculation ranged from 50 to 100 μm. This is in accordance with observations of the hepatic stages of the same strain of *P. falciparum* (NF54) in chimpanzee livers (average diameter of 60 to 100 μm on day-6 post-inoculation)^39^. It is interesting to note that when different lines of *P. falciparum* were used, a Rumanian strain in humans^40^ or a Liberian strain in chimpanzees^41^, the average sizes on days 6 were 60 and 35 μm, respectively. Given that these are the only *in vivo* observations made on the liver stages of *P. falciparum*, it is not possible to speculate on the nature of the size variations observed. On the other hand, it is clear that hepatic parasite maturation is slower in *in vitro*-cultured human primary hepatocytes than in hepatocytes engrafted in the immune-compromised mice^5^. Moreover, the uninucleate forms that are usually observed in *in vitro* culture of the *P. falciparum* hepatic stages^23^, presumably arrested forms, were not observed in any of the liver sections taken from the FRG^19^ or the TK-NOG mice. Such arrested or retarded forms had not been reported from previous *in vivo* observations, which re-enforces the quality and suitability of the hepatic environment for parasite development in the LH mice. The speed of maturation of the *P. ovale* hepatic parasites in the TK-NOG mice (average diameter of 65 μm on day 8) was also closer to that observed *in vivo*, where schizont sizes ranged from an average of 45 μm on day 5 to 75 μm on day 9 in humans^32^, and of 40 μm on day 8 in chimpanzee livers^34^. It is interesting to note that for this species, the speed of maturation *in vitro*^36^ appears to mirror that observed, albeit on a limited number of occasions, in the human or chimpanzee hosts.

The detection of *P. falciparum* antigens expressed in the asexual blood stages (SERP) or in merozoites (MSP1_19_, AMA-1 and RON-4) strongly indicated that full maturation of the hepatic schizonts to yield invasive merozoites was obtained in the TK-NOG mice. This was further supported by the observation of merosome-like structures, merosomes being packets of merozoites that had been previously reported in the rodent malaria parasites as the means by which their hepatic merozoites reach the blood stream on hepatic schizont rupture^29^,^30^. Our observations of merosome-like structures in the livers of *P. falciparum*-infected TK-NOG mice duplicate those made in FRG mice^19^, indicating that this strategy of parasite egress from the infected hepatocyte might be characteristic of all *Plasmodium* species. Incontrovertible proof that infectious *P. falciparum* merozoites were generated from the infected engrafted hHEP was obtained in double-engrafted TK-NOG mice (with hRBC). Thus, blood samples taken from all eight mice in three independent experiments (engrafted with distinct hHEP and infected with different *P. falciparum* sporozoite batches) were positive for blood stage parasites shortly after *in vitro* cultivation and, more importantly, four out of four mice from two of the experiments cited above developed a microscopically detectable blood parasitaemia from days 8 or 9 post-sporozoite inoculation, which was maintained over many weeks of follow-up. This transition was also reported in FRG mice^19^, although in that case, the blood stages could only be demonstrated through *in vitro* cultivation of total blood collected on day 7 post-inoculation in mice that had received injections of hRBC the previous day as well as 2 hours before killing.

To date, long-term maintenance of *P. falciparum* blood parasitaemias had been obtained in mice engrafted solely with hRBC that were: (a) inoculated with high numbers of infected RBC^15^; and/or (b) that were under additional immunosuppressive treatment (chlodronate-liposomes to eliminate macrophages, antibodies to deplete polymorphonuclear leukocytes)^12^,^16^,^17^,^42^; and/or (c) that were inoculated with a strain of *P. falciparum* that had been adapted to mice^15^,^43^. The presence of gametocytes was only reported in some of these previous studies^12^,^16^,^17^. The double-engrafted TK-NOG mice (with hHEP and hRBC) made it possible to overcome the limitations of previous models, such as the need to adapt the *P. falciparum* strain to the mice^15^,^43^ or the necessity for continuous treatment with clodronate liposome to deplete macrophages^16^, and thus proved suitable to obtain all the stages of the *P. falciparum* life cycle. In particular, the TK-NOG mice offered an environment conducive to the production and maturation of the sexual blood stages. Indeed immature gametocytes were observed starting from day-26 post-sporozoite inoculation, with mature stage V forms gradually appearing throughout the follow-up period. The total gametocyte numbers could account for up to 8.5% of all the parasites forms observed, mature gametocytes reaching up to 2.5% of the circulating parasites. Similar to previous observations in other mouse strains^12^,^16^,^17^, gametocytes of all stages of maturity were observed in the mice's peripheral circulation, a situation that sharply contrasts with the situation in humans where maturation is completed in the bone marrow and the spleen before the release of the infective gametocytes to the periphery and eventual transmission to mosquitoes^44^. It would be of interest to elucidate the reasons for this difference. Recently, the full life cycle of *P. falciparum* was reported in humanized HLA-DR4.RagKO.IL2RγcKO.NOD (DRAG) mice^45^ transplanted with CD34+ haematopoietic stem cells. Parasitaemia levels, where measured, did not exceed a few parasites per μl, and efficient transmission to mosquitoes required prior *in vitro* cultivation although direct transmission from one infected mouse was reported. Furthermore, the value of immunocompromised mice models for the study of relapsing parasites has been strongly enhanced by the recent successful demonstration of the development of *P. vivax* hepatic stages, including hypnozoites, in FRG KO mice engrafted with human primary hepatocytes^46^.

The TK-NOG model used in our study has been recently shown to represent a suitable model for preclinical drug validations^47^,^48^. Here we show that this extends to investigations of the *Plasmodium* species that infect humans. The main advantages of the TK-NOG model are the freedom from additional treatment post-engraftment, the high levels and sustainability of the humanized compartments, and the potential for double engraftment that made it possible to obtain consistently all the stages of the *P. falciparum* life cycle within the same animal directly from a sporozoite inoculation. This makes it possible to envisage routine *in vivo* preclinical validation of compounds targeting *P. falciparum* liver stage, genetic crosses of *P. falciparum* strains that had become severely limited by the necessity to employ chimpanzees, and *in vivo* validation of *P. falciparum* attenuated live vaccines targeting the liver stages. In addition, obtaining mature *P. falciparum* gametocytes allows to envisage the use of this model for transmission to mosquitoes, and, if validated, for *in vivo* evaluation of transmission blocking interventions. Finally, the success in obtaining hHEP infected with sporozoites from two distinct *P. ovale* strains directly obtained from clinical isolates and observations of viable late-developing forms, probably activated hypnozoites, makes the TK-NOG model suitable for *in vivo* investigations of relapsing *Plasmodium* species hitherto restricted to primate models.

## Methods

### Ethics statement

This study was carried out in strict accordance with the Guide for the Care and Use of Laboratory Animals from the Central Institute for Experimental Animals (CIEA) in Japan and with the French and European regulations (2010/63/EU). The experimental protocols were approved by the Animal Care Committee of the CIEA (Permit Number: 11029A) and by the Ministère de l′Education Nationale, de l'Enseignement Supérieur et de la Recherche (Authorization Number 01737.03). Human blood samples infected with *P. ovale* were obtained from adult after receiving written informed consent. The collection and use of this material was approved by the Institutional Review Board (Comité de Protection des Personnes) of the Centre Hospitalo-Universitaire Pitié-Salpêtrière, Assistance Publique-Hôpitaux de Paris, Paris, France.

### Liver- and RBC-humanization of TK-NOG mice

TK-NOG mice were imported from Dr Hiroshi Suemizu's laboratory (CIEA, Japan) and bred at the Centre d'Expérimentation Fonctionnelle (CEF, La Pitié-Salpêtrière, Paris) under strict pathogen-free conditions. Given that transgenic males are hypofertile, non-transgenic males were bred with transgenic females, progeny was genotyped and transgenic males were engrafted with hHEP as described by Hasegawa *et al.*^20^. Only transgenic males were used for further hHEP engraftment because the higher expression of the HSVtk transgene in males as compared with that in females (Supplementary Fig. 5) allowed higher level of endogenous hepatocyte lysis and, subsequently, more efficient human hepatocyte engraftment. Briefly, 7–9 week-old transgenic males were injected with gancyclovir and lysis of endogenous hepatocytes was evaluated through alanine amino transferase (ALT) measurement on 32 μl of heparinised plasma using a Reflovet chemistry analyzer (ScilVet, France) at the CEF (La Pitié-Salpêtrière, Paris). When ALT level exceeded 100 IU l^−1^, mice were injected intrasplenically with 1 million thawed hHEP previously isolated and frozen in the lab from a 60-year-old male patient negative for HIV, HCV and HBV^23^. Efficiency of hHEP engraftment was then evaluated every month by quantifying human albumin within mouse plasma with the Human Albumin ELISA Quantitation Set (Bethyl laboratories) following manufacturer recommendations. Hasegawa *et al.*^20^ previously showed that hAlb plasmatic level correlated with replacement index . Gancyclovir was not administered after transplantation.

Mice used for the present study were either bred and LH in the CEF as mentioned above, or imported already engrafted with commercial hHEP from H. Suemizu's laboratory (Supplementary Table S2), and had human albumin level ranging from 1 to 10 mg ml^−1^, corresponding to 15–90% of humanization^20^. For *P. falciparum* full life cycle experiments, LH TK-NOG mice showing hAlb levels≥5 mg ml^−1^ were selected for further engraftment with hRBC. hRBC group O were obtained from the Etablissement Français du Sang blood bank. Mice were injected daily through the i.p. route with 1 ml of hRBC, 50% haematocrit in RPMI medium, following a protocol adapted from Angulo-Barturen *et al.*^15^, starting 6 days before sporozoite injection. Volume 0.5 μl of blood was taken at the tail vein to monitor hRBC engraftment by flow cytometry. Engraftment with hRBC was maintained up to 6 weeks post-sporozoite injection.

### Sporozoites and *in vivo* infection

*P. falciparum* (NF54 strain) sporozoites were isolated by aseptic dissection of the salivary glands of infected *A. stephensi* obtained from the Department of Medical Microbiology, University Medical Centre, St Radboud, Nijmegen, The Netherlands. Anaesthetised mice were injected intravenously into the retro-orbital sinus vein with 1–3.5 million sporozoites. *P. ovale* sporozoites were obtained from infected *A. stephensi* salivary glands after a blood meal on *P. ovale*-infected blood. *P. ovale* species diagnosis was performed at the Service de Maladies Infectieuses, Centre National de Référence pour le Paludisme, Laveran Building, La Pitié-Salpêtrière, by microscopic examination of thick and thin blood smears by a highly experienced technician and was further confirmed by PCR^49^. Fresh heparinized blood from *P. ovale*-infected patients containing gametocytes was used to feed mosquitoes. Briefly, plasma and buffy coat were removed by centrifugation, pelleted RBC were then washed twice with RPMI and resuspended in human AB plasma at 50% haematocrit. All of this process was done at 37 °C to maintain gametocytes infectivity. Volume of 2.5 ml was used to fill a membrane-based feeder system and mosquitoes were fed for 1 h. Mosquitoes were then kept at 27 °C. Stomachs were analysed at day 8 post-infected blood meal to check for the presence of oocysts. Salivary glands were dissected on day 14 post-blood feeding for sporozoite isolation. Anaesthetised mice were injected intravenously into the retro-orbital sinus vein with sporozoites. Mice were inoculated with *Plasmodium* sporozoites at least 2 months after the gancyclovir treatment. Mice were killed at different days post-sporozoite injection. After removal of the gall bladder, the liver was cut into pieces and fixed in formalin for paraffin inclusion, or snap-frozen in liquid nitrogen before storage at −80 °C. Snap-frozen samples were further processed for immunohistology analysis on liver sections.

### Follow-up of P. falciparum blood stages *in vivo* and *in vitro*

*P. falciparum* blood stage infection in liver- and RBC-humanized mice was monitored starting from day-8 post-sporozoite injection on thick and thin blood smears, done with, respectively, 2 and 4 μl of fresh blood collected at the tail on EDTA. At day 8 post-sporozoite injection, 200 μl of blood was taken at the retro-orbital sinus vein under anaesthesia for *in vitro* culture of blood stage parasites. Briefly, heparinized blood was washed twice with RMPI medium containing 0.5% albumax (Gibco), 20 μg ml^−1^ of gentamycin (Gibco), 0.23% of sodium bicarbonate (Gibco), and 0.1 mM Hypoxanthine (Sigma), and diluted half with fresh O hRBC for further culture in flask at 5% haematocrit, in a 5% CO2 humid atmosphere. Medium was renewed every 48 h and 2-μl thick or thin blood smears were made to monitor blood-stage parasite emergence and growth.

Fixed-permeabilized thick and thin blood smears were GIEMSA-stained before analysis for quantitation of asexual and sexual blood stages. Results were either expressed as the number of asexual/sexual forms per μl of blood after complete examination of thick blood smears, or percentage of parasitaemia estimated after analysis of at least 50 fields, magnification × 50, on thin blood smears (number of infected RBC × 100/total number of infected and uninfected RBC).

### Detection of hepatic parasites in liver sections

Formalin-fixed liver samples were embedded in paraffin and processed at the Anatomo-Pathology Service of the Pitié-Salpêtrière Hospital, Paris. Briefly, automated paraffin inclusion was done in cassettes, 5-μm thick serial sections were cut with a microtome, deparaffinized and either stained with Hematoxylin-Eosin-Safran at the Anatomo-Pathology Service, or processed for *Plasmodium* HSP70 staining at the laboratory. In this latter case, antigen retrieval was performed by incubating sections in a pH8 - 1 mM EDTA buffer at 98 °C for 30 min, followed by 20 min at room temperature to allow samples temperature to cool down, and then washed twice in PBS before processing for HSP70 immunostaining as described below for frozen sections.

Sixteen- to fifty-μm thick serial sections of frozen liver samples were made with a Cryostat HM500, air dried for 30 or 60 min before fixation in methanol for 5 min or 4% PFA for 20 min at room temperature. Sections were then washed twice in PBS before processing for HSP70 immunostaining. Briefly, 5-μm thick deparaffinised sections and 16-μm thick frozen sections were first incubated with normal goat serum diluted 1:500 for 1 h, washed twice in PBS, incubated with a mouse polyclonal anti-*Plasmodium* HSP70 serum diluted 1:1,000 (ref. 50) for 1 h, washed twice and finally incubated with a goat anti-mouse IgG coupled to Alexa-Fluor 488 or to Alexa-Fluor 594 diluted 1:500 (Life Technologies) and DAPI 1 μg ml^−1^ in the dark for 45 min. All incubations were done in PBS-1%BSA-0.2%Triton X-100 and at 37 °C. Staining with anti-Pf EXP-1 (1:200, ref. 24), SERP (1:400, ref. 25), AMA-1 (rat mAb 4G2, 1:100, ref. 27), MSP1_19_ (1:50, ref. 51), RON-4 (mouse mAb 24C6 4F12, 1:100, ref. 28) and anti-human Aquaporin-9 (goat mAb C18, Santa Cruz Biotechnology, 1:40) were performed following the protocol described above, using either goat anti-mouse, goat anti-rabbit, goat anti-rat or donkey anti-goat IgG coupled to Alexa-Fluor 488 or Alexa-Fluor 594 diluted 1:500 (Life Technologies) as secondary antibodies. For parasite immunostaining in 50-μm thick frozen sections, incubation with the anti-*Plasmodium* HSP70 immune serum was performed overnight at room temperature and all incubations were done in PBS-1%BSA-0.2%Triton X-100. All sections were washed twice in PBS before being mounted in anti-fading medium and stored at 4 °C before analysis. Immunostained sections were examined under a fluorescence microscope (Leica DMI 4000 B). Photomicrographs were obtained using a confocal microscope (Olympus FV-1000, Plateforme d'Imagerie Cellulaire PICPS, La Pitié-Salpêtrière, Paris) and images were analysed using ImageJ software. Whole-slide imaging was done using a digital slide scanner Nanozoomer 2.0 HT equipped with the fluorescent unit option L11600-05 and a 3-CCD TDI camera using a x20 objective (Hamamatsu Photonics, Plateforme d'Imagerie, Institut de la Vision, Paris). Scanned images were analysed with the NDPView Nanozoomer associated software.

### *In vivo* hRBC monitoring by flow cytometry

Volume of 0.5 μl of EDTA-blood were stained with anti-human-Glycophorin A mAb coupled to FITC (eBiosciences), washed in PBS, fixed in 0.2% PFA and analysed using a LSR Fortessa cytometer (Plateforme de cytométrie en flux CYPS, La Pitié-Salpêtrière, Paris). Analyses were performed using the FlowJo software 7.6.3.

### Statistical analyses

The sizes and areas of *P. falciparum* schizonts at days 5 and 7 were compared using the Mann–Whitney *U-*test (GraphPad Prism software).

## Additional information

**How to cite this article:** Soulard, V. *et al. Plasmodium falciparum* full life cycle and *Plasmodium ovale* liver stages in humanized mice. *Nat. Commun.* 6:7690 doi: 10.1038/ncomms8690 (2015).

## Supplementary Material

Supplementary Information Supplementary Figures 1-5 and Supplementary Table 1
