# Supplementary material for: Plasmodium falciparum full life cycle and Plasmodium ovale liver stages in humanized mice
Source: Nat Commun. 2015 Jul 24;6:7690. doi: 10.1038/ncomms8690 (PMC4525212; doi:10.1038/ncomms8690)
Supplement: Supplementary Information — Supplementary Figures 1-5 and Supplementary Table 1 [file ncomms8690-s1.pdf]

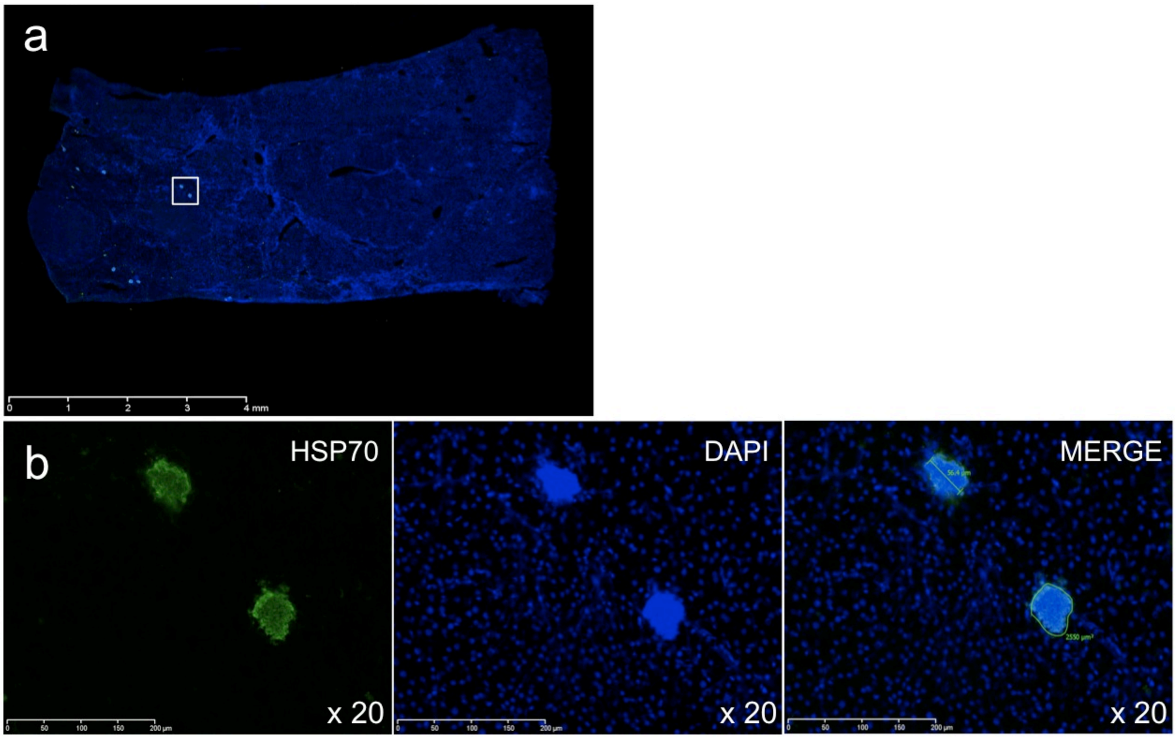

Supplementary figure 1 : Medium throughput analysis of *P. falciparum* hepatic development through the use of a fluorescence slide scanner.

50 µm-thick liver sections were stained for *Plasmodium* HSP70 and DAPI and analyzed with a fluorescence slide scanner. **A.** Picture of a whole liver section stained for HSP70 and DAPI showing numerous day 7-schizonts. **B.** Magnification x20 of two mature schizonts.

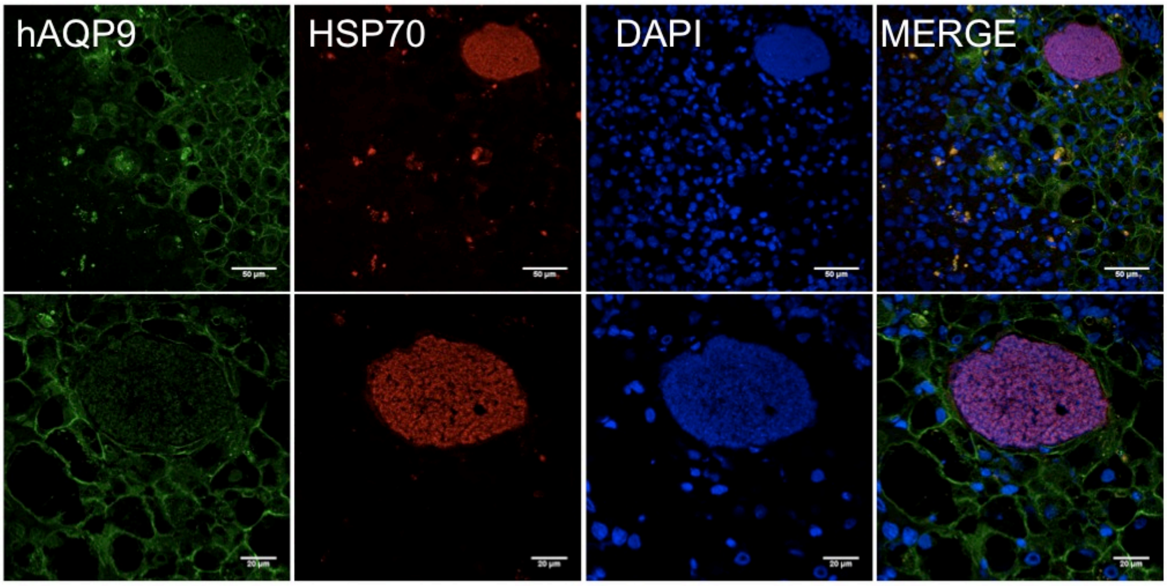

Supplementary figure 2: *P. falciparum* develops within engrafted hHEP in LH TK-NOG mice.

Triple staining for human membrane AQP9 protein (green), *Plasmodium* HSP70 (red) and DAPI (blue) showing parasite growth within human engrafted hepatocyte. Upper panel, bar = 50 µm. The lower panel is a higher magnification of the schizont showed above, bar = 20 µm.

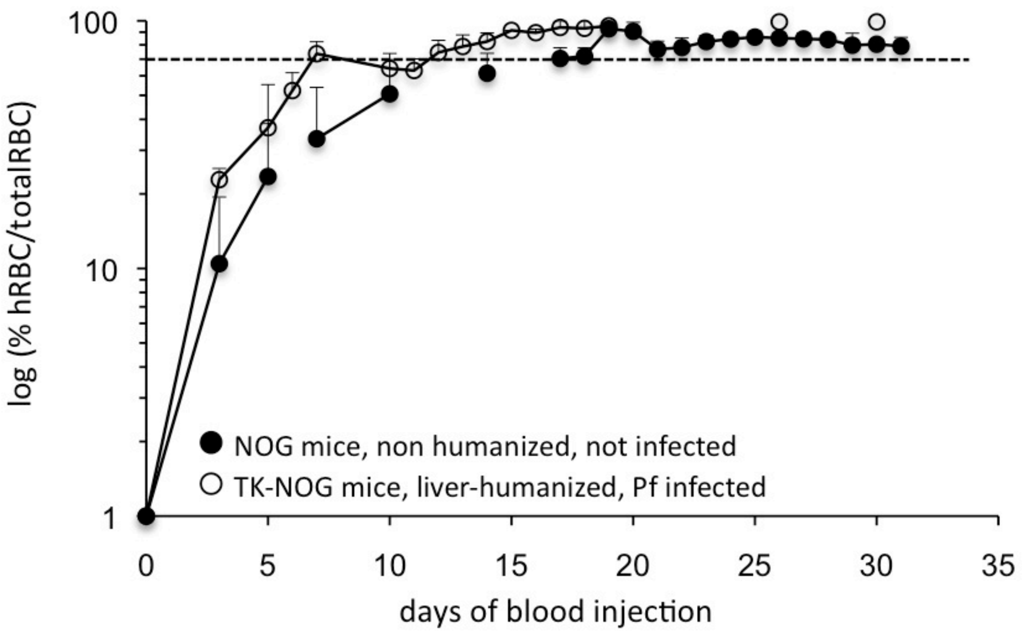

Supplementary figure 3 : hRBC engraftment in TK-NOG mice.

TK-NOG non transgenic mice (NOG mice), non humanized and not infected (n = 4 mice), and LH-TK NOG mice, infected with *P. falciparum* sporozoites at day 6 of hRBC injection (n = 8, 3 independent experiments), were daily injected with hRBC and the percentage of hRBC in the peripheral blood was monitored by flow cytometry. Results are expressed as the percentage of hRBC among total RBC (mean ± SD). The dashed line shows 70% of humanization.

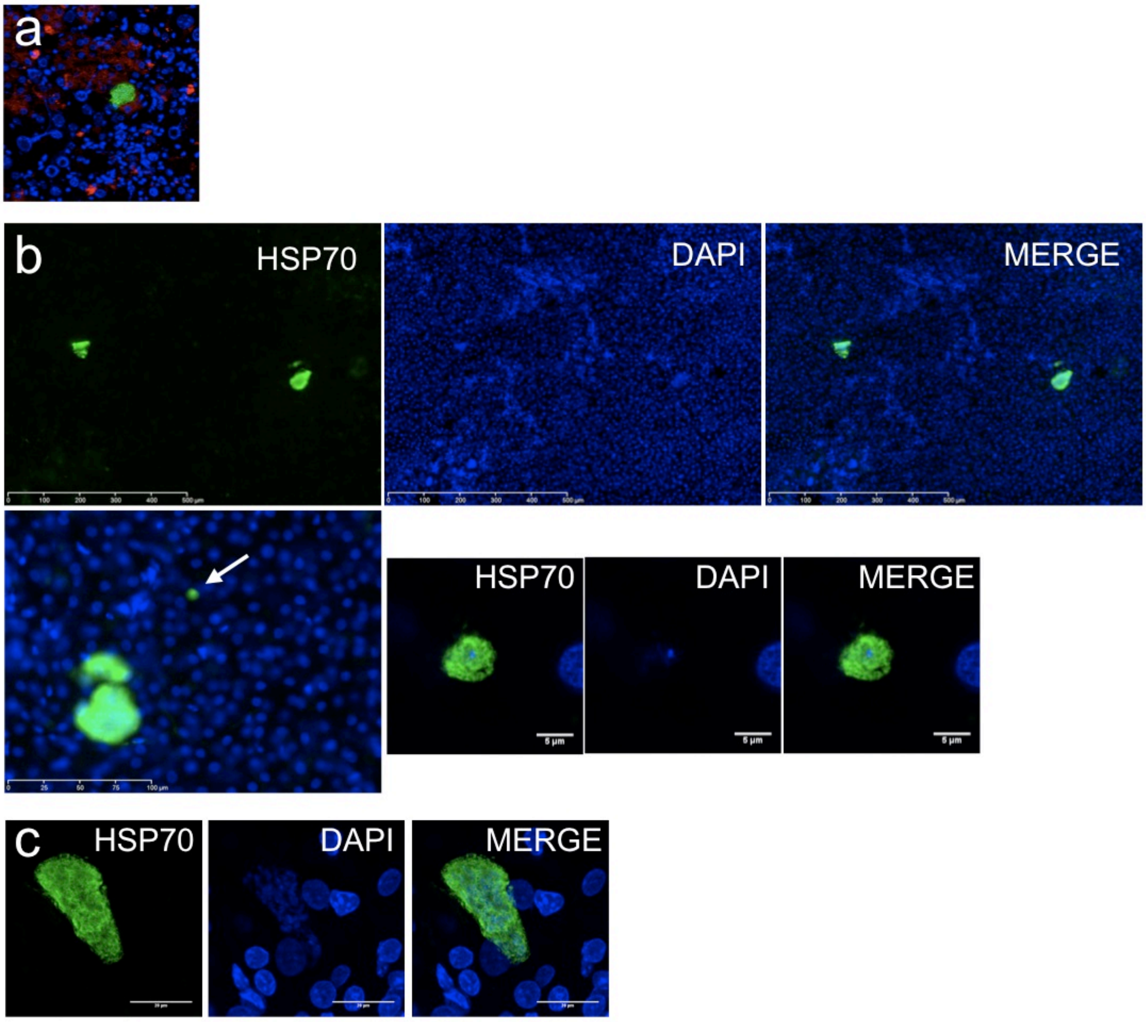

Supplementary figure 4 : *P. ovale* hepatic development in LH-TK NOG mice.

Representative pictures of *P. ovale* schizonts and hypnozoites at days 8 and 21 of infection stained by indirect immunofluorescence on 16- and 50 µm-thick frozen liver sections. **A.** Triple staining for *Plasmodium* HSP70 (green), human albumine (red) and DAPI (blue) showing parasite growth within human engrafted hepatocyte; bar = 20 µm. **B.** Fluorescent slide scanner pictures showing two *P. ovale* mature schizonts (upper panel), and a schizont close to a hypnozoite (white arrow, left picture of lower panel). Lower panel, right pictures: higher magnification of the hypnozoite showing the unique nucleus of the round shaped 5 µm diameter parasite; bar = 5 µm. C. Picture of the day 21-schizont found in the first *P. ovale* infection; bar = 20 µm.

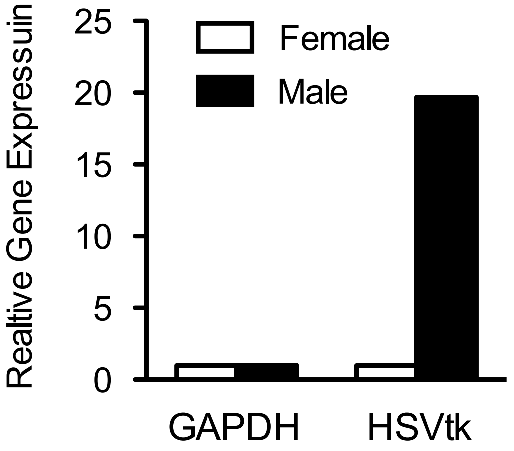

Supplementary Figure 5: Gender difference in transgene expression.

Comparison of HSVtk transgene expression between female and male TK-NOG mice of same age (8 weeks old) by RT-qPCR. The expression of HSVtk transgene was normalized to the expression of glyceraldehyde 3-phosphate dehydrogenase (GAPDH).

	Mouse #	hAlb (mg/mL)[a]	%hRBC[b]
**Exp #1**	1†	5.2	84.6
	2	5.1	77.8
**Exp #2**	3†	5.7	82.7
	4†	7.4	97.9
	5†	9.8	98.1
**Exp #3**	6	9.3	83.7
	7	5.5	93.4
	9	7.2	98.2

Supplementary Table 1: Levels of liver- and RBC-humanization in doubly engrafted TK-NOG mice.

[a]Level of human albumin within two weeks before starting engraftment of hRBC, one week before inoculation of sporozoites.

[b]Percentage of hRBC measured between day 6 and 8 post-sporozoite inoculation.

All three experiments were performed with distinct batches of human hepatocytes, human RBC and *P. falciparum* sporozoites.

†Mice #1, 3, 4 and 5 were monitored for parasitemia *in vivo*.

	Batch	Gender	Age
**Lonza CC-2591S[a]**	7F3063	F	4 Y
**BPI HEP187[a]**	HEP187266-TA05	M	5 Y

Supplementary Table 2: Human hepatocytes used for engrafment of TK-NOG mice in CIEA. [a] Negative for HIV, HBV, HCV
